# Cobalamin Deficiency in Children and Adolescents with Sickle Cell Disease

**DOI:** 10.3390/nu17030597

**Published:** 2025-02-06

**Authors:** Dunia Hatabah, Rachel Krieger, Lou Ann Brown, Frank Harris, Rawan Korman, Loretta Reyes, Jasmine Umana, Laura Benedit, Bridget A. Wynn, Chris A. Rees, Carlton Dampier, Claudia R. Morris

**Affiliations:** 1 Department of Pediatrics, Emory University School of Medicine, Atlanta, GA 30322, USA; dunia.hatabah@emory.edu (D.H.); r.krieger90@gmail.com (R.K.); lbrow03@emory.edu (L.A.B.); fharris@emory.edu (F.H.); rawan.korman@emory.edu (R.K.); loretta.reyes@emory.edu (L.R.); bridget.wynn@emory.edu (B.A.W.); chris.rees@emory.edu (C.A.R.); cdampie@emory.edu (C.D.); 2 Children’s Healthcare of Atlanta, Atlanta, GA 30329, USA; laura.benedit@choa.edu; 3 Children’s Hospital of Philadelphia, Philadelphia, PA 19104, USA; umanaj@chop.edu

**Keywords:** sickle cell disease, cobalamin, B_12_, nitrous oxide

## Abstract

**Background/Objective**: Cobalamin (B_12_) deficiency is reported in 18% of adults with sickle cell disease (SCD) and only 10% without SCD; limited data are available on children. Diagnosing B_12_ deficiency is challenging given the lack of an established gold standard method of assessment and the unique renal features of SCD. B_12_ metabolism can be impacted by the clinical use of nitrous oxide gas (N_2_O), which is a standard therapy for SCD pain in some European countries. In response to emerging reports of neurologic sequalae in patients with SCD receiving N_2_O, we evaluated the prevalence of B_12_ deficiency in children with SCD pain. **Methods:** Secondary analysis of prospective blood and urine samples in children aged 3–21 hospitalized with SCD pain. B_12_ deficiency was defined as plasma methylmalonic acid (MMA) > 592 nmol/L or urine MMA/creatinine ≥ 2.2 mmol/mol. **Results:** Ninety-four children (13 ± 4 years, 54% female, 68% hemoglobin-SS, and 72% on hydroxyurea) were assessed. Further, 53% (50/94) had B_12_ deficiency diagnosed by either urine, plasma, or both; 27% (25/94) were deficient based on urine; 39% (37/94) were deficient by plasma; and 13% (12/94) were deficient by both plasma and urine. Plasma MMA and urine MMA/creatinine did not correlate with hemoglobin or mean corpuscular volume. **Conclusions:** B_12_ deficiency was common in children with SCD. The absence of a gold standard for diagnosing B_12_ deficiency compounded with the reliability issues of testing modalities make it impractical to determine whether this is an over- or under-estimation of the true prevalence. Future studies to better understand the dynamics of B_12_ metabolism during acute and steady states in SCD are warranted and could elucidate the influence of acute SCD pain on these biomarkers.

## 1. Introduction

Cobalamin (B_12_) deficiency has been reported in 18% of adults with sickle cell disease (SCD) but in only 10% without SCD [1]; limited data are available on children. SCD may create a unique risk for B_12_ deficiency as a result of high basal metabolic rates, erythrocyte turnover, and hemolysis, which contribute to both macro- and micronutrient deficiencies [2].

**Figure 1:**
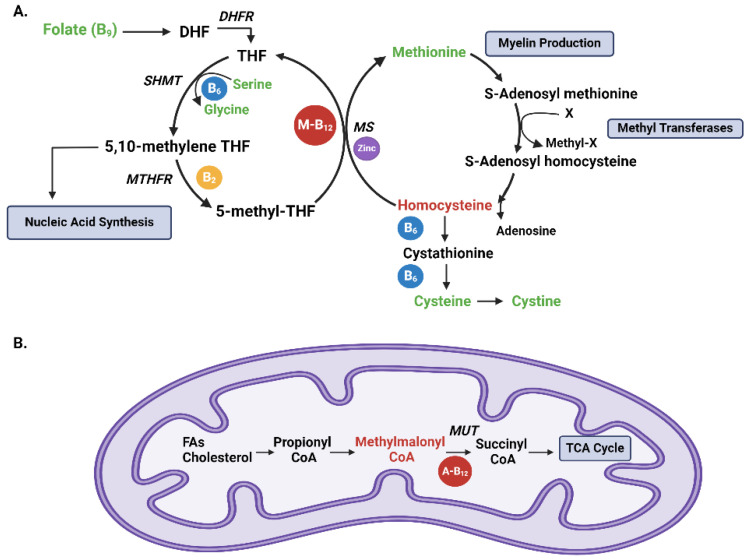
Vitamin B_12_-dependent reactions (**A**) [3,8], B vitamin metabolism. The folate cycle starts with folate (B_9_) conversion into dihydrofolate (DHF), which is subsequently reduced into tetrahydrofolate (THF) by the enzyme dihydrofolate reductase (*DHFR*). Serine hydroxy methyltransferase (*SHMT*) then converts THF to 5,10-methylene THF, coupled to the hydroxylation of serine to glycine utilizing vitamin B_6_ (blue circle). 5,10-methylene THF is important for nucleic acid synthesis. 5,10-methylene THF is then reduced to 5-methyl THF by methylene tetrahydrofolate reductase (*MTHFR*) using vitamin B_2_ (yellow circle). Methionine synthase (*MS*) dependent on vitamin B_12_ in the form of methylcobalamin (M-B12; red circle) and zinc (purple circle) utilize the methyl group from 5-methyl THF to produce methionine from homocysteine. Methionine is important for myelin production. Methionine, in turn, can generate S-adenosyl methionine, an important methyl donor, donating a methyl group to methyltransferases and becoming S-adenosyl homocysteine, which is then recycled into homocysteine. Methyltransferases are involved in biological methylation reactions, including RNA, DNA, histone, and protein methylation. Homocysteine can then be converted to cystathionine and to cysteine, which is in turn converted to cystine. Items in green are all part of our amino acid analysis. Items in red are markers for B_12_ deficiency. (**B**) B12 and metabolism of propionate. Branched-chain and odd-chain fatty acids (FAs) and cholesterol side chains are all metabolized to be used in the tricarboxylic acid cycle (TCA). They are initially converted to propionyl CoA, which is then converted to methylmalonyl CoA. Methylmalonyl CoA is then converted to succinyl CoA via methylmalonyl CoA mutase (MUT), a vitamin B_12_-dependent reaction utilizing adenosyl B_12_ (A-B_12_; red circle). Succinyl CoA enters the TCA cycle [9].

Cobalamin plays a key role in various metabolic pathways within the human body. Its primary mechanism of action involves its participation as a cofactor in two key reactions: the conversion of homocysteine to methionine (Figure 1A), and the conversion of methylmalonyl-CoA to succinyl-CoA [3] (Figure 1B). These reactions are essential for DNA synthesis, red blood cell formation, and the production of myelin, the protective sheath around nerve fibers [3,4,5,6,7].

Symptoms of cobalamin deficiency vary from mild fatigue to severe irreversible neurologic impairment. Megaloblastic anemia is the classic consequence of B_12_, folate deficiency, or both, with a characteristically high mean corpuscular volume (MCV). Neurologic symptoms of B_12_ deficiency arise due to disturbances in myelin production. Neuropsychiatric symptoms can be the initial and even the sole manifestation of B_12_ deficiency [10]. Given that patients with SCD have higher rates of neuropsychiatric problems, an underlying B_12_ deficiency in this population may be easily missed.

The lack of an established gold standard method of assessment compounded with an absence of data to guide cobalamin deficiency screening in SCD makes the diagnosis of B_12_ deficiency challenging. Measurement of plasma B_12_ is known to be inaccurate, yet it is commonly used by clinicians as a screening tool. Methylmalonic acid (MMA) and homocysteine are both substrates of B_12_-dependent enzymatic reactions **(**Figure 1A,B) and are elevated in the plasma of >90% of B_12_-deficient patients [11,12]. However, homocysteine is also elevated in multiple vitamin B deficiencies, including folic acid, vitamin B_6_ (pyridoxine), and vitamin B_2_ (riboflavin), and is not as specific or sensitive as MMA in diagnosing B_12_ deficiency (Figure 1A) [13,14,15]. MMA can be measured in both plasma or urine however, plasma MMA accumulates in settings of renal dysfunction [16] and may become unreliable in B_12_ deficiency diagnosis among those with impaired renal function. Therefore, urine MMA-to-urine creatinine (MMA/Cr) ratio has been suggested as an alternative for the diagnosis of B_12_ deficiency to account for poor renal function [17,18] however, the influence that the defects of glomerular hyperfiltration that occur early in the course of SCD have on both MMA clearance and plasma accumulation is unknown. Therefore, in the setting of SCD and its unique renal impairment, the reliability of both plasma and urine biomarkers of B_12_ deficiency may be further compromised. At this time, there are no established data to guide best practices for the screening and diagnosis of B_12_ deficiency in patients with SCD.

The growing use of nitrous oxide gas (N_2_O) in the United States, especially for dental procedures and in some institutions for SCD pain treatment, adds to the urgency of addressing the risk of B_12_ deficiency in patients with SCD. N_2_O is an inhaled medication that is anxiolytic, with mild-to-moderate analgesic properties [19]. Although generally considered safe [20], patients can experience serious neurologic complications from N_2_O inhalation as it adversely impacts cobalamin metabolism [21,22,23]. N_2_O is used as an analgesic for SCD vaso-occlusive pain episodes (VOEs) in several European countries, with over 70% of French emergency departments using a nitrous oxide/oxygen mix for the management of SCD pain [24]. Unfortunately, several case reports of neurological sequelae due to N_2_O use in patients with SCD have been reported in France and Britain [21,25,26,27], and the risks are not universally appreciated by emergency medicine physicians.

In response to this emerging problem, we assessed the prevalence of B_12_ deficiency diagnosed either by plasma MMA or urine MMA-to-urine creatinine ratio (MMA/Cr) in a cohort of children and adolescents with SCD, hospitalized for vaso-occlusive pain episodes (VOEs) after seeking care in a pediatric emergency department (ED).

## 2. Materials and Methods

This is a secondary analysis of samples collected prospectively from a single-center pharmacokinetic/pharmacodynamic study (NCT02447874) and a phase 2 randomized controlled trial (NCT02536170), both evaluating mechanisms of hemolysis in acute SCD pain. Blood and urine samples were collected from children aged 3–21 years with SCD seeking care at the ED and who were then hospitalized for vaso-occlusive pain and received parenteral opioids.

Samples from enrolled patients were collected either in the ED before the patients were hospitalized (with an ED admission order placed) or within 12 hours of admission. All patients had plasma MMA, plasma amino acids, and urine MMA analyzed via high-pressure liquid chromatography-linked tandem mass spectrometry and urine creatinine measured via enzyme-linked immunosorbent assay (ELISA). Subgroup analysis of plasma folic acid and holotranscobalmin was conducted, utilizing ELISA on 37 patients who had elevated plasma MMA values. Demographic information, sickle cell genotype, hydroxyurea status, history of folic acid supplementation, complete blood count, reticulocyte percentage, and complete metabolic panel data were extracted from electronic medical records. Estimated glomerular filtration rate (eGFR) was calculated for all patients with height measurements using the Bedside Schwartz formula [28,29,30].

Methylmalonic acid concentrations in the plasma and urine were determined by liquid chromatography—mass spectrometry/mass spectrometry using a Force C18 column, as outlined by the manufacturer (Restek Corporation^®^; Bellefonte, PA, USA) [31]. Mobile phase A was 0.5% formic acid in water and mobile phase B was 0.5% formic acid in methanol. Samples were spiked with MMA-D3 proteins precipitated with 0.5% formic acid in methanol, and the supernatant filtered using a standard filter vial prior to analysis. Commercially available MMA (Restek Corporation^®^; Bellefonte, PA, USA) was used to establish lower and upper limits of detection as well as a standard curve. The lower limit of detection for plasma MMA was 592 nmol/L and was used as the threshold to determine B_12_ deficiency. For urine, MMA was expressed relative to creatine with MMA/Cr ≥ 2.2 mmol/mol (2.3 mg/g) used to define B_12_ deficiency (based on Quest Diagnostics standard cut off) [32]. Severe B_12_ deficiency was defined as MMA/Cr > 5. MMA/Cr < 2.2 was considered a normal B_12_ status, and MMA/Cr of 1.8–2.2 indicated possible B_12_ deficiency.

Assuming a baseline prevalence of at least 18% [1] for B_12_ deficiency in children with SCD-VOE, a sample size of 90 provided ≥ 80% power to detect a difference of ≥8% (assuming a baseline prevalence of ≤10% B12 deficiency in non-SCD patients based on adult reported data with a significance level of 0.05).

The Pearson’s correlation coefficient was used to assess linear relationships between continuous variables. Differences between continuous variables amongst groups were assessed using the unpaired Students *t*-test, whereas Pearson’s Chi-squared (or Fisher’s exact when appropriate) test was used to compare categorical variables across groups. The inter-test reliability of deficiency status was assessed between urine and plasma samples through percent agreement analysis. Positive percent agreement (PPA) and negative percent agreement (NPA) were performed to measure the agreement of test positivity/sensitivity and test negativity/specificity detected by the urine and plasma tests.

## 3. Results

A total of 94 children were included (Table 1). The mean age was 13 ± 4 years and 54% were female. Among the included patients, 68% had hemoglobin-SS and 72% were on hydroxyurea.

Of the 94 patients, 25 (27%) had vitamin B_12_ deficiency with MMA/Cr ≥ 2.2, of whom 5 had severe vitamin B_12_ deficiency (MMA/Cr > 5). Six patients had MMA/Cr between 1.8 and 2.1, which reflected a possible B_12_ insufficiency. Further, 37 (39%) patients were diagnosed with B_12_ deficiency by plasma MMA and 12 patients (13%) were diagnosed by both plasma MMA and urine MMA/Cr.

There were no significant differences in patient demographics and clinical characteristics across all groups (Table 1). There was a slight male predominance in patients diagnosed with MMA/Cr by urine versus a female predominance in patients diagnosed by plasma MMA. There were significant differences in urine creatinine (*p* < 0.0001), urine MMA (*p* < 0.0001), MMA/Cr (*p* < 0.0001), and plasma MMA (*p* = 0.02) between deficient and sufficient groups diagnosed by urine MMA/Cr. There were no significant differences between deficient and sufficient groups diagnosed by plasma MMA. In addition, there were significant differences (*p* < 0.001) in MMA/Cr, urine creatinine (mg/dL), and urine MMA between deficient groups diagnosed by plasma MMA vs. deficient and sufficient groups diagnosed by urine MMA/Cr. There were no significant differences in plasma levels of holotranscobalamin, folic acid, or all amino acids across all groups (Supplementary Table S1).

Both MMA/Cr and plasma MMA did not significantly correlate to classic hematologic markers of B_12_ deficiency, including hemoglobin, hematocrit, and mean corpuscular volume (MCV). Additionally, both markers did not correlate to plasma homocysteine, holotranscobalamin, or folic acid levels. Plasma MMA did not correlate to urine MMA/Cr across all groups; plasma creatinine did not correlate to urine creatinine.

Agreement in B_12_ deficient vs. B_12_ sufficient status when analyzing urine versus plasma data was present in 56/94 of the patients, with an overall diagnostic accuracy of 60% (Table 2). The PPA was 28%, with 12 patients diagnosed with B_12_ deficiency by both plasma and urine (Table 2 and Table 3).

## 4. Discussion

In this study of children with SCD-VOE, more than half had B_12_ deficiency diagnosed by either urine MMA/Cr, plasma MMA, or both. The prevalence identified in this study is considerably higher than what has been previously reported in children with SCD [33,34,35,36,37].

While a higher prevalence of B_12_ deficiency was formerly recognized in adults with SCD [1,38,39], a recent study out of Tanzania found consistently low cobalamin levels across different age groups [40]. A limitation of many studies of cobalamin status is the misguided use of serum B_12_, an unreliable marker, as the diagnostic marker of B_12_ deficiency [41]. Serum B_12_ measurement is suboptimal since it can remain within the normal range in patients with clinical and biological symptoms of B_12_ deficiency [42,43]. Additionally, serum B_12_ can be influenced by various factors which confound the interpretation of its measurement. For example, serum B_12_ is elevated in several conditions relevant to SCD including hemolysis, liver disease, and small intestinal bacterial overgrowth, in addition to myeloproliferative diseases, thereby masking B_12_ deficiency [41].

While serum B_12_ is known to be unreliable, plasma MMA has consistently outperformed other diagnostic tests for B_12_ deficiency [41]. However, plasma MMA may become inaccurate in the diagnosis of B_12_ deficiency in those with kidney impairment because it accumulates during renal dysfunction. The urinary MMA-to-urinary creatinine ratio accounts for renal disease [17]; however, the impact that the unique renal impairment of hyperfiltration specifically in young patients with SCD [44] has on this ratio is unknown. Surprisingly, urine MMA and urine MMA/Cr did not correlate to plasma MMA in our pediatric cohort, in contrast to previous reports in non-SCD patients [45,46]. In addition, when comparing the results of the two diagnostic tests, a percent agreement of 60% was observed, indicating moderate discrepancy between the tests. The observed discrepancy in diagnostic concordance raises important questions about the context of VOEs. Vaso-occlusive pain episodes are associated with significant physiological stress and metabolic disturbances, which may influence the clearance, production, or excretion of MMA. While the cause of this observed discrepancy is unknown, we speculate that both VOEs and unique renal dysfunction in patients with SCD may be a contributing factor. Children diagnosed with B_12_ deficiency by urine MMA/Cr had high urinary MMA and low urinary creatinine compared to B_12_-sufficient groups who had lower urinary MMA and higher urinary creatinine (Table 1). MMA is excreted efficiently by the kidneys [46] and is concentrated in the urine, making it a sensitive marker of tissue depletion. Children with SCD can experience glomerular hyperfiltration and hyposthenuria as early manifestations of kidney disease [47]. Glomerular hyperfiltration can result in a higher urinary excretion of both MMA and creatinine, but the impact of an impaired urine concentrating capacity on the urinary concentrations of both MMA and creatinine has not been adequately studied. Urinary B_12_ status may be influenced by the presence of underlying kidney dysfunction in the deficient population, but other markers of kidney dysfunction are needed. Adding to this is the observed sex-related difference across groups, with a predominance of males in the patients diagnosed by urine compared to a female predominance in the group diagnosed by plasma. Given that sex-based differences in renal function have been reported in patients with SCD [48], our data suggest that sex may contribute to variability in B_12_ metabolism and renal function in patients with SCD.

The lack of a definitive gold standard for diagnosing B_12_ deficiency specifically in SCD is further compounded by the unreliability of typical hematologic parameters to help identify a cobalamin (or folate) deficiency. The characteristic MCV elevation that develops with B_12_ deficiency is not easily identified in patients with SCD due to MCV elevation from hydroxyurea use [49] and folic acid supplementation. In our cohort, cobalamin deficiency diagnosed by either plasma MMA or MMA/Cr did not correlate to MCV or hemoglobin, most likely because most of our patients were on both hydroxyurea and folic acid. In addition, other markers of B_12_ deficiency described in the literature, such as holotranscobalamin and homocysteine, did not correlate to either plasma MMA or MMA/Cr. This suggests a possible difference in B_12_ metabolism in patients with SCD compared to individuals without SCD. Over-supplementation with folate may account for the lack of homocysteine elevation in these patients, as homocysteine is dependent on both folic acid and B_12_ [50,51].

Empiric folate supplementation is the standard practice for children with SCD, owing theoretically to a higher demand for hematopoiesis despite limited evidence to support this practice [52]. The biologic activity of supplemental folate is dependent on the hepatic enzyme dihydrofolate reductase, ultimately methylating folate to its biologically active form 5-methyl tetrahydrofolate [53]. However, dihydrofolate reductase is unusually slow in humans, and unmetabolized folic acid has been detected in the umbilical cord of fetuses, infant blood, and in children with SCD supplemented with prophylactic high-dose folate (1 mg/day) [54]. While the effects of unmetabolized folic acid are not well established, it has been speculated to influence B_12_ metabolism, folate metabolism, DNA methylation, and gene expression [55]. Supplementation with naturally existing, biologically active forms of folates such as folinic acid (Leucovorin) or 5-methyl tetrahydrofolate may be considered superior to folic acid as they can function even in the face of defective folate metabolism. However, it is important to note that neither folic acid nor cobalamin can work independently without each other and are dependent on other B vitamins and micronutrients such as B_2_, B_6_, and zinc—which is also a mandatory cofactor for methionine synthase. It is likely more beneficial for patients with SCD to be supplemented with a combination of these essential vitamins/micronutrients rather than any one in isolation.

The consequences of B_12_ deficiency assume additional risk among this demographic due to greater use of N_2_O gas outside of the operating room in the United States, in dental procedures [56], in the emergence department for pain [19], and possibly for priapism [57]. Although N_2_O is generally considered safe, it is associated with neurologic and neuropsychiatric manifestations due to cobalamin inhibition, which decreases methionine levels important for myelin production [58]. N_2_O is a standard therapy for sickle cell-related pain in some European countries including France [24], and reports of irreversible neurological sequelae in patients with SCD and undiagnosed B_12_ deficiency who received frequent N_2_O treatment are emerging [21,25,26,27]. In addition to neurologic complications, acute anemia and hyperhemolysis with elevated levels of lactate dehydrogenase have been reported in patients with SCD receiving N_2_O gas, associated with undetectable B_12_ levels [27]. Anemia improved rapidly after the discontinuation of N_2_O and administration of oral B_12_. Subclinical B_12_ deficiency may also contribute to isolated episodes of hyperhemolysis in patients with SCD and acute pain [59]. While we cannot establish whether B_12_ deficiency in children with SCD is acute or chronic from this study, the combination of a possible, transient, VOE-related disruption in B_12_ metabolism and the potential effects of N_2_O may heighten the risk of deficiency during hospitalization. Recognizing and understanding these interactions is crucial.

This study has several limitations. First, this is a single-center study with a relatively small sample size, which may limit the generalizability of our findings. Additionally, the assay available in our laboratory for plasma MMA analysis had a limit of detection ≥ 592 nmol/L due to the limited sample volume available for plasma MMA analysis. The diagnostic threshold for the clinical assessment of B_12_ deficiency based on the reference range from Quest Diagnostics is 318 nmol/L. To measure values in the range of the Quest references, a more sensitive assay would be needed, or a larger sample volume, which were not available. Hence, it is plausible that some truly deficient patients may have been missed. Our study also assessed kidney function via serum creatinine, which is widely used for assessing renal function, but there are several limitations to its use in eGFR calculations that could potentially impact cobalamin status interpretation. In patients with SCD, glomerular hyperfiltration and proximal tubule dysfunction can impact both the filtration and secretion of creatinine, with subsequent over-estimation of kidney function [60]. Creatinine also varies by muscle mass; therefore, it is sex-dependent due to the higher muscle mass in males. The utilization of other markers such as cystatin c is increasing, including in patients with SCD, but is not yet standard clinical practice. Additional markers of renal function may be useful in the determination of B_12_ deficiency in SCD populations and requires further investigation. Another limitation is the absence of corroborating clinical data reflective of symptoms of B_12_ deficiency not collected in this study, which could have further supported the diagnoses. However, it is important to note that the symptoms of B_12_ deficiency—such as fatigue, pallor, irritability, and neurological changes—overlap significantly with the chronic manifestations of SCD, particularly anemia and neuropsychiatric symptoms. This overlap can mask or delay the recognition of B_12_ deficiency in SCD patients, leading to underdiagnosis and undertreatment. Finally, samples were collected when patients were undergoing an acute pain event, and the potential impact of VOEs on parameters such as plasma MMA, urine MMA, urine creatinine, or urine MMA/Cr are unknown. Therefore, we cannot say with certainty that the observed B_12_ assessments accurately reflect the patients’ B_12_ status in the absence of VOEs.

This study addressed a clinically relevant gap in the knowledge on B_12_ status in children with SCD. The major strength of our study is the use of both plasma MMA and urine MMA/Cr as markers of B_12_ deficiency, which allowed for a more comprehensive assessment of B_12_ deficiency, potentially capturing a broader spectrum of patients that could have been otherwise overlooked. Notably we are the first group to assess cobalamin status using urine MMA/Cr in children with SCD, which may hold promise as a non-invasive sampling technique that is convenient and feasible in pediatric populations.

## 5. Conclusions

Our data confirm that B_12_ deficiency is common in children with SCD. Given the challenges in the reliability of current testing modalities, it is not possible to determine whether this is an over- or under-estimation of the true prevalence.

Given the consequences and increased risk of B_12_ deficiency in SCD, compounded with the increased clinical use of N_2_O gas in the United States and parts of Europe, future studies are needed to better understand the dynamics of B_12_ metabolism during acute and steady states of SCD. Longitudinal studies that assess plasma and urinary MMA levels in both steady state and during VOEs could elucidate the influence of acute events on these biomarkers. Additionally, research investigating the prevalence, causes, and clinical consequences of B_12_ deficiency in children with SCD is critical. Such studies could guide the development of standardized diagnostic criteria and inform targeted interventions to improve nutritional and overall health outcomes in this population.

## Figures and Tables

**Table 1:** Demographics, clinical characteristics, and markers of B_12_ deficiency comparison between patients diagnosed by urine methylmalonic acid/creatinine (MMA/Cr), plasma MMA, or both.

Variable	Overall n = 94	Diagnosis by Urine MMA/Cr		Diagnosis by Plasma MMA	Diagnosis by Urine and Plasma
		B_12_ Deficient n = 25	B_12_ Sufficient n = 69	B_12_ Deficient n = 37	B_12_ Deficient n = 12
**Mean age, years (SD**)	13 ± 4	13 ± 4	13 ± 5	13 ± 3	13 ± 3
**Sex n (%)**					
Female	51 (54%)	12 (48%)	39 (57%)	24 (65%)	9 (75%)
Male	43 (46%)	13 (52%)	30 (43%)	13 (35%)	3 (25%)
**Genotype**					
Hb SS	64 (68%)	18 (72%)	47 (67%)	22 (59%)	8 (67%)
Hb SC	14 (15%)	3 (12%)	11 (15%)	4 (11%)	2 (17%)
HbS-β^+^Thal	7 (7%)	1 (4%)	6 (9%)	6 (17%)	1 (8%)
HbS-β^0^Thal	9 (10%)	3 (12%)	6 (9%)	5 (13%)	1 (8%)
**Clinical Labs**					
Hemoglobin (g/dL)	9 ± 2	9 ± 1	9 ± 2	9 ± 2	9 ± 1
Hematocrit (%)	27 ± 5	26 ± 4	27 ± 5	26 ± 5	26 ± 4
MCV (fL)	86 ± 13	87 ± 13	87 ± 13	84 ± 12	83 ± 11
Reticulocytes (%)	10 ± 5	12 ± 5	10 ± 5	10 ± 5	12 ± 6
ALT (IU/L)	27 ± 12	28 ± 12	28 ± 12	27 ± 12	30 ± 13
AST (IU/L)	48 ± 24	56 ± 32	46 ± 21	49 ± 28	64 ± 37
Total bilirubin (mg/dL)	3 ± 2	3 ± 3	3 ± 2	3 ± 2	3 ± 3
BUN (mg/dL)	7 ± 3	8 ± 3	7 ± 2	7 ± 2	6 ± 3
Creatinine (mg/dL)	0.5 ± 0.1	0.5 ± 0.2	0.5 ± 0.2	0.5 ± 0.1	0.5 ± 0.1
eGFR	134 ± 39	126 ± 28	137 ± 42	133 ± 35	120 ± 25
**SCD Medications**					
Hydroxyurea, **Yes** n (%)	68 (72.3%)	20 (80%)	47 (68%)	28 (76%)	9 (75%)
Folic acid, **Yes** n (%)	87 (92.5%)	23 (92%)	64 (93%)	32 (86%)	11 (91%)
**Markers of B_12_ deficiency**					
Urine creatinine (mg/dL)	48 ± 26	25 ± 9 *	57 ± 25 *	43 ± 22 **	26 ± 6
Urine MMA (ng/mL)	626 ± 314	950 ± 380 *	508 ± 177 *	647 ± 286 **	912 ± 263
Urine MMA/Cr (mg/g)	1.8 ± 1.6	4.0 ± 1.7 *	1.0 ± 0.5 *	1.9 ± 1.5 **	3.7 ± 1.3

No statistically significant differences between B_12_-sufficient and B_12_-deficient subjects diagnosed by plasma MMA. * *p* < 0.0001; ** statistically significant differences between B_12_-deficient groups diagnosed by plasma MMA and both sufficient and deficient groups diagnosed by urine MMA/Cr. MCV: mean corpuscular volume; AST: aspartate amino transferase; ALT: alanine amino transferase; BUN: blood urea nitrogen; eGFR: estimated glomerular filtration rate; MMA: methylmalonic acid; Cr: creatinine.

**Table 2:** Percent agreement analysis between urine and plasma samples, assessing inter-test reliability of deficiency status, showing diagnostic estimators and Wilson confidence intervals (CIs).

Statistic	Estimator	Lower 95% CI	Upper 95% CI
PPA	0.480	0.300	0.665
NPA	0.638	0.520	0.741
PPV	0.324	0.196	0.485
NPV	0.772	0.648	0.862
Overall Diagnostic Accuracy	0.596	0.495	0.689

PPA: positive percent agreement; NPA: negative percent agreement; PPV: positive predictive value; NPV: negative predictive value.

**Table 3:** Number of patients with B_12_ deficiency diagnosed by either plasma MMA, urine MMA/Cr, or both.

Diagnosis	Urine Deficient	Urine Sufficient	Total
**Plasma Deficient**	12	25	37
**Plasma Sufficient**	13	44	57
**Total**	25	69	94

## Data Availability

Data are contained within the article or Supplementary Materials.

## Supplementary Materials

The following supporting information can be downloaded at www.mdpi.com/10.3390/nu17030597/s1, Table S1: Folic acid, holotranscobalamin and amino acid levels in patients diagnosed by urine methylmalonic acid/creatinine (MMA/Cr), plasma MMA or both.

## Abbreviations

The following abbreviations are used in this manuscript:

B12	Cobalamin
SCD	Sickle Cell Disease
MMA	Methylmalonic acid
MCV	Mean Corpuscular Volume
N_2_O	Nitrous Oxide Gas
Cr	Creatinine
ED	Emergency Department
eGFR	Estimated Glomerular Filtration Rate
VOE	Vaso-occlusive Pain Episodes
PPA	Percent Positive Agreement
NPA	Negative Percent Agreement
AST	Aspartate Aminotransferase
ALT	Alanine Aminotransferase
BUN	Blood Urea Nitrogen
