# Supplementary material for: Cobalamin Deficiency in Children and Adolescents with Sickle Cell Disease
Source: Nutrients. 2025 Feb 6;17(3):597. doi: 10.3390/nu17030597 (PMC11819659; doi:10.3390/nu17030597)
Supplement: Supplementary file 1 [file nutrients-17-00597-s001.zip › nutrients-3453043-supplementary.pdf]

Supplementary Table S1: Folic acid, holotranscobalamin and amino acid levels in patients diagnosed by urine methylmalonic acid/creatinine (MMA/Cr), plasma MMA or both.

		Diagnosis by urine MMA/Cr		Diagnosis by plasma MMA		Diagnosis by urine and plasma
**Variable**	**Overall n= 94**	**B_{12} Deficient n= 25**	**B_{12} Sufficient n= 69**	**B_{12} Deficient n= 37**	**B_{12} Sufficient n= 57**	**B_{12} Deficient n= 12**
Folic Acid (nM)	60 ± 17	58 ± 18*	62 ± 17**	60 ± 17	N/A	58 ± 18
Holotranscobalamin (pM)	302 ± 109	332 ± 86*	288 ± 118**	302 ± 110	N/A	332 ± 86
Methionine(μM)	21 ± 6	20 ± 6	22 ± 6	22 ± 6	21 ± 6	19 ± 5
Serine(μM)	20 ± 8	17 ± 5	20 ± 8	19 ± 6	20 ± 9	17 ± 5
Glycine(μM)	53 ± 26	50 ± 15	54 ± 29	52 ± 17	54 ± 30	50 ± 15
Cysteine(μM)	708 ± 380	787 ± 531	679 ± 309	697 ± 326	713 ± 415	608 ± 311
Cystine(μM)	9 ± 8	8 ± 7	9 ± 8	9 ± 8	9 ± 8	6 ± 5
Homocysteine(μM)	7 ± 12	7 ± 18	5 ± 4	5 ± 4	8 ± 15	5 ± 4

There we no statistically significant differences between all groups.
Folic Acid and Holotranscobalamin were only assessed in patients with elevated plasma MMA (n=37)
*n=12
** n=25
